# High intensity intermittent games-based activity and adolescents’ cognition: moderating effect of physical fitness

**DOI:** 10.1186/s12889-018-5514-6

**Published:** 2018-05-08

**Authors:** Simon B. Cooper, Karah J. Dring, John G. Morris, Caroline Sunderland, Stephan Bandelow, Mary E. Nevill

**Affiliations:** 1 Exercise and Health Research Group, Sport, Health and Performance Enhancement (SHAPE) Research Centre, Department of Sport Science, Nottingham Trent University, Nottingham, NG11 8NS UK; 2 School of Sport, Exercise and Health Sciences, Loughborough University, Loughborough, UK

**Keywords:** Team games, Executive function, Working memory, Young people, Maximal oxygen uptake

## Abstract

**Background:**

An acute bout of exercise elicits a beneficial effect on subsequent cognitive function in adolescents. The effect of games-based activity, an ecologically valid and attractive exercise model for young people, remains unknown; as does the moderating effect of fitness on the acute exercise-cognition relationship. Therefore, the aim of the present study was to examine the effect of games-based activity on subsequent cognition in adolescents, and the moderating effect of fitness on this relationship.

**Methods:**

Following ethical approval, 39 adolescents (12.3 ± 0.7 year) completed an exercise and resting trial in a counterbalanced, randomised crossover design. During familiarisation, participants completed a multi-stage fitness test to predict VO_2_ peak. The exercise trial consisted of 60-min games-based activity (basketball), during which heart rate was 158 ± 11 beats∙min^− 1^. A battery of cognitive function tests (Stroop test, Sternberg paradigm, trail making and d2 tests) were completed 30-min before, immediately following and 45-min following the basketball.

**Results:**

Response times on the complex level of the Stroop test were enhanced both immediately (*p* = 0.021) and 45-min (*p* = 0.035) post-exercise, and response times on the five item level of the Sternberg paradigm were enhanced immediately post-exercise (*p* = 0.023). There were no effects on the time taken to complete the trail making test or any outcome of the d2 test. In particular, response times were enhanced in the fitter adolescents 45-min post-exercise on both levels of the Stroop test (simple, *p* = 0.005; complex, *p* = 0.040) and on the three item level of the Sternberg paradigm immediately (*p* = 0.017) and 45-min (*p* = 0.008) post-exercise.

**Conclusions:**

Games-based activity enhanced executive function and working memory scanning speed in adolescents, an effect particularly evident in fitter adolescents, whilst the high intensity intermittent nature of games-based activity may be too demanding for less fit children.

## Background

An acute bout of exercise has been shown to have beneficial effects on cognitive function in adults [1] and children [2, 3], with the effect generally reported as being small but positive [1, 3]. Furthermore, although less work has been conducted in adolescents (11–18 years old), there is evidence to suggest that an acute bout of exercise also enhances cognition in this population [4–7]. However, there are a number of moderating variables that may influence the effect of exercise on cognition, including the intensity and modality of exercise, and the components of cognitive function examined [1, 8]. Furthermore, age is considered a key factor in the exercise-cognition relationship [1, 9] and thus findings from other populations cannot easily be translated to adolescents, where cognitive function is of particular importance for academic achievement [10, 11].

The majority of the literature examining the acute effects of exercise on cognitive function in adolescents has focused upon more traditional continuous exercise models, including running [4] and cycling [6]. However, the evidence suggests that activity patterns in adolescents are typically high intensity and intermittent in nature and very rarely consist of sustained moderate or vigorous intensity activity [12], with 95% of ‘bouts’ of physical activity being less than 15 s in duration [13]. Therefore, it is important that the effects of such high intensity intermittent activity on cognition are examined. In this regard, it has recently been shown that 10 × 10 s sprints (with 50 s active recovery between sprints) enhances the speed of executive function in adolescents [5]. However, the efficacy and safety of high intensity intermittent exercise training in this population remains questionable.

The latest data suggest that only 27% of young people (aged 5–18 years) in the United States [14] and 21% of boys and 16% of girls in the United Kingdom [15] meet the current recommendations of 60 min moderate to vigorous physical activity per day, which has been implicated in the relatively high (~ 30%) prevalence of overweight and obesity in these populations [15, 16]. Adolescence is also the age at which physical activity levels typically see the sharpest decline, which is of concern given not only the cognitive benefits that exist, but also the more well-documented beneficial effects on physical health, psychosocial health and the social benefits gained [17].

Furthermore, the effectiveness of structured opportunities for physical activity in young people has been questioned [18], with young people typically selecting to participate in activities that they enjoy [19]. Therefore, perhaps games-based activity provides a more attractive exercise model for adolescents, eliciting the high intensity intermittent activity patterns but in a more acceptable and enjoyable manner [20]. To date, only one study has examined the effect of team games-based activity on cognition in adolescents, reporting greater free recall memory following exercise, particularly when the exercise consisted of games-based activities [21]. However there was no pre-exercise baseline measurement of cognitive function in this study, making it difficult to discern the effects of the exercise. There is also limited evidence regarding the effects of games-based activity in younger children. Interestingly, Gallotta et al. [22] reported that basketball-based activity led to smaller improvements in attention (assessed by the d2 test) when compared to aerobic circuit training, suggesting that games-based activity did not optimise subsequent cognition in children. Conversely, Ishihara et al. [23] reported that playing tennis resulted in greater improvements in executive function than repetitive exercise and a technique-based approach in 6–11 year old children. However, this study employed a cross-sectional design and thus confounding variables may have affected study outcomes.

Therefore, the effects of games-based activity on cognitive function in young people are not clear. It is possible that the discrepancies in the literature are due to the different age groups studied, with the combined physical and mental exertion in team games ‘overloading’ the younger children in the study of Gallotta et al. [22], whilst proving beneficial for the adolescents in the study of Pesce et al. [21]. Furthermore, it could also be due to the different components of cognitive function examined, in line with previous suggestions that the effects of exercise on cognition may be domain specific [1, 2]. Therefore, it is important for future studies to assess the effects of games-based exercise on a range of cognitive domains in adolescents to further explore this, especially given games-based activity is a mode of exercise that adolescents enjoy and chose to take part in [19].

Also of interest is the effect of physical fitness on cognitive function and academic achievement. Using a cross-sectional study design, higher levels of physical fitness (as assessed using a 20 m shuttle run test) have been associated with greater academic achievement [24]. Further exploring this relationship, higher levels of physical fitness have been associated with enhanced performance across a range of cognitive domains, including memory [25, 26], attention [27] and executive function [7, 26]. Furthermore, Crova et al. [28] reported that executive function was greater in fitter 9–10 year old children (as assessed by a 20 m shuttle run test), but that the acute effect of exercise (although beneficial) was not affected by the fitness level of the children.

The only study to date to examine the moderating effect of fitness on the acute exercise-cognition relationship was performed in young adults aged 23 years and used a simple one item physical activity questionnaire to categorise participants according to activity and fitness levels [29]. The study reported that an acute bout of exercise only enhanced attention in those with higher levels of physical fitness [29]. However, the exercise protocol in this study was a maximal exercise test to exhaustion and thus the fitter participants completed exercise of a greater duration and intensity; thus, it is not possible to distinguish the effects of this from the effects of physical fitness. Therefore, the moderating effect of fitness on the acute exercise-cognition relationship warrants further investigation, particularly in adolescents where it has not been studied to date.

Therefore, the aim of the present study was two-fold: (1) to examine the acute effects of a bout of games-based activity across a range of domains of cognitive function in adolescents; and (2) to examine whether physical fitness (predicted VO_2_ peak from a 20 m shuttle run test) moderates the acute effect of games-based activity on cognitive function in this population. Based on the literature to date we hypothesise that games-based activity will enhance adolescent’s cognitive function and tentatively propose that this effect may be more pronounced in higher fit adolescents.

## Methods

### Participant characteristics

Forty-one young people (aged 11–13 years) were recruited to participate in the study. However, two participants failed to complete the study because they were absent from school for one of the experimental trials. Therefore, a total of 39 participants completed the study (20 male, 19 female). Anthropometric measures of height, body mass, sitting height, waist circumference and skinfolds were taken for descriptive purposes. Height was measured using a Leicester Height Measure (Seca, Germany) accurate to 0.1 cm and body mass was measured using a Seca 770 digital scale (Seca, Germany) accurate to 0.1 kg, allowing the determination of body mass index (BMI). Sitting height was measured and subsequently leg length was calculated to allow the estimation of maturity (by calculating years from peak height velocity) using the method of Moore et al. [30].

### Study design

Following approval from the institutions ethical advisory committee, participants were recruited from two secondary schools in the East Midlands, UK. In accordance with the guidelines for school-based research, head teacher consent was gained in addition to written informed consent from parents/guardians and a health screen questionnaire completed for each participant. Participants provided their assent to participate in the study on each testing day.

The study employed a randomised crossover design, with participants blind to the condition until arrival at school for the first main experimental trial. Participants completed a familiarisation trial and two main experimental trials (exercise and resting), each separated by 7 days, thus participants acted as their own controls. During familiarisation the protocol of the study was explained to participants and they were provided an opportunity to practice and become familiar with the procedures to be used during the study. This information was also provided to parents/guardians prior to the study via both written information and a phone call from an experimenter. Opportunities were provided for participants/parents/guardians to ask questions to clarify any aspect of the study they did not fully understand.

**Fig. 1:**
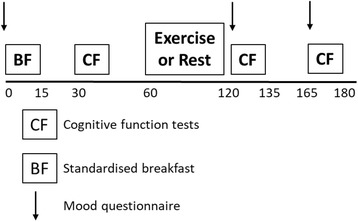
Experimental Protocol

In brief, the main experimental trials followed a 2-day protocol (Fig. 1). On day one, following baseline measurements a standardised breakfast (identical to the breakfast of Cooper et al. [4]) was provided, to control for the potential of breakfast and exercise to interact and affect cognition in young people [31]. Sixty minutes following breakfast, participants completed 60 min of basketball (exercise trial) or continued to rest (resting trial). A battery of cognitive function tests and a mood questionnaire was completed 30 min pre-, 5 min post- and 45 min post-exercise. Other procedures during the study included capillary blood samples, blood pressure measurement and the provision of a standardised lunch. The aim of these measures was to assess the effects of exercise on cardio-metabolic risk factors in young people, the results of which are to be presented elsewhere. This paper will present and discuss the findings with regards to cognitive function. The experimental protocol is shown in Fig. 1.

### Pre-trial control

Participants consumed a meal of their choice the evening before each main experimental trial and repeated this for the subsequent trial, following which they fasted from 10 pm until arrival at school the following day. Water was allowed ad libitum during this time to maintain euhydration. Participants were also asked to avoid any exercise for 24 h prior to each experimental trial. A telephone call was made to parents/guardians prior to each experimental trial to remind them of this information. All participants followed the pre-trial conditions.

### Exercise and rest protocol

The exercise protocol consisted of 60 min of basketball activity, completed in a school sports hall in groups of 8–10 participants. The basketball sessions consisted of a warm-up, skill-based drills and small sided games. All sessions were run by the same experienced level 4 basketball coach. Participants were also familiarised with the basketball session during the familiarisation trial. During the resting trial (and at all times during the exercise trial, with the exception of the 60 min basketball session) participants rested quietly whilst seated in a school classroom. Throughout both experimental trials heart rate was monitored continuously using the Firstbeat team sport system (Firstbeat Technologies Ltd., Finland).

### Cognitive function tests

The battery of cognitive function tests lasted approximately 12 min and consisted of the Stroop test, Sternberg paradigm, trail making test and d2 test of attention, with tests completed in that order. Three of the tests (Stroop test, Sternberg paradigm and trail making test) were completed on a laptop computer (Lenovo Thinkpad T450; Lenovo, Hong Kong) whilst the d2 test was administered via paper and pencil. The instructions for each cognitive function test were presented prior to each completion of the test and participants were allowed an opportunity to ask any questions. Each test (and test level) was preceded by a number of practice stimuli to re-familiarise participants with the test and negate any potential learning effects, the data for which were discarded. Participants completed the tests in a classroom of 8–12 participants, in silence and separated such that participants could not interact during the completion of the tests. Participants also wore sound cancelling headphones to minimise external disturbances. This testing procedure has been previously used successfully in a similar study population [4, 5, 31].

#### Stroop test

The Stroop test is a commonly used measure of selective attention and executive function, in particular the domain of inhibitory control [32]. The Stroop test consisted of two levels (simple and complex). On both levels, a test word is placed in the centre of the screen with the target and distractor presented randomly on the left and right side of the screen, with participants selecting their response using the appropriate arrow key. The simple level contained 20 stimuli, with the test word, target and distractor all presented in white font. The complex (colour-interference) level contained 40 stimuli, with the participant selecting the colour the word was written in rather than the actual word (an incongruent colour). On both levels, participants were asked to respond as quickly and accurately as possible. The choices remained on the screen until the participant responded, with an inter-stimulus interval of 1 s. The variables of interest were the response time of correct responses and the proportion of correct responses made.

#### Sternberg paradigm

The Sternberg paradigm [33] is a commonly used test of working memory and consisted of three levels, each using a different working memory load (one, three or five items). On the one item level the target was always the number ‘3’, consisting of 16 stimuli, whereas on the three and five item levels the target was three or five randomly generated letters respectively, each containing 32 stimuli. At the start of each level the target items were displayed along with instructions to press the right arrow key if a target item was presented and the left arrow key for a distractor. The correct response was counterbalanced between the left and right arrow key for each level and on all levels, the choice stimuli was presented in the centre of the screen with an inter-stimulus interval of 1 s. The variables of interest were the response time of correct responses and the proportion of correct responses made.

#### Trail making test

The trail making test is a commonly used measure of information processing speed [34] and consisted of two levels, each containing 25 randomly distributed stimuli. On each level participants were asked to click on the targets in sequence. For the number level, the sequence was sequential numbers (i.e. 1, 2, 3 etc.) and on the number and letter level the sequence was alternating numbers and letters (i.e. 1, A, 2, B etc.). The variable of interest was the time taken to complete each level.

#### d2 test of attention

The d2 test of attention [35] is a paper and pencil letter cancellation test, used to assess selective attention. The test consists of 14 lines of 47 letters (either ‘d’ or ‘p’) with 0–2 dashes above and below each letter. Participants were asked to mark any letter ‘d’ with 2 dashes (i.e. 2 above, 2 below or 1 above and 1 below). Participants were allowed 20 s to complete each line, following which an experimenter instructed them to move on to the next line. The first 2 lines of the test were for practice/re-familiarisation and the data were discarded. Each test was then coded by an investigator for the total number of items processed (n), errors of omission (number of d’s with 2 dashes missed; EO) and errors of commission (any distractor items incorrectly marked; EC). This allowed the calculation of three outcome variables: Concentration Performance (CP) = n-EO-EC; Processed Targets (PT) = (n-EO) + EC; and error percentage (E%) = (EO + EC)/PT. These variables were used for subsequent analysis.

### Mood questionnaire

The mood questionnaire was a modified version of the ‘Activation-Deactivation Check List’ (AD ACL) short form [36] which has been used successfully in a similar study population [4, 5, 32]. The 20 item questionnaire was split into four components of mood; energy, tiredness, tension and calmness, each having five corresponding adjectives on the questionnaire. Participants were asked to respond to a series of adjectives regarding how they felt at that moment in time, on a scale of 1 to 5 (where 1: definitely do not feel, 3: unsure, 5: definitely feel). The scores on the adjectives for each component of mood were summed, providing an overall score for each component. In addition, three visual analogue scales were used to provide a measure of participants’ hunger, fullness and concentration. The scales consisted of a 10 cm line from one extreme to the other (i.e. not at all to very), with participants indicating the point on the line that applied to them at that moment in time.

### Fitness measurement

In order to provide an assessment of cardiorespiratory fitness, participants completed the multi-stage fitness test [37] to predict VO_2_ peak (ml∙kg^-1^∙min^− 1^). The multi-stage fitness test required participants to complete progressive 20 m shuttle runs in time with an audio signal, until the point of volitional exhaustion or until they could not maintain the required running speed to keep time with the audio signal. The test commenced at a speed of 8.5 km∙h^− 1^ and increased by 0.5 km∙h^− 1^ for every stage completed. Investigators verbally encouraged participants throughout the test to encourage all participants to work to the point of volitional exhaustion. Predicted VO_2_ peak was calculated using an adolescent specific calculation [38] and heart rate was monitored throughout the test using the previously described methods.

### Statistical analysis

Data from the Stroop test and Sternberg paradigm were analysed using R (www.r-project.org). Response time analyses were performed using the nlme package, which implements mixed effect models and yields *t* statistics. Accuracy analyses were performed using the lme4 package, which implements mixed effect models for data with a binomial outcome distribution and yields *z* statistics. Initially, analyses were conducted using a two-way (trial by time) interaction for each test level given that each level requires a different level of cognitive processing. For all variables from the trail making test, d2 test of attention and mood questionnaire, analyses were conducted using SPSS (version 24; SPSS Inc., Chicago, IL., USA) using a two-way (trial by time) repeated measures analysis of variance (ANOVA).

Furthermore, to assess the moderating effect of fitness on the exercise-cognition relationship, participants were assigned to high (top 50% for each sex) and low (bottom 50% for each sex) fitness groups, based on the predicted VO_2_ peak from the multi-stage fitness test. For each variable from the cognitive function tests a three-way (trial by time by fitness) interaction was then conducted using mixed effect models in R (Stroop test and Sternberg paradigm) or repeated measures ANOVA in SPSS (trail making test and d2 test of attention), with fitness as a between subjects factor. Heart rate was compared between fitness groups using independent samples t-tests and mood data assessed using a mixed methods ANOVA. All data are presented as mean ± standard error of the mean (SEM) and for all analyses, statistical significance was accepted as *p* < 0.05.

## Results

### Participant characteristics

The characteristics of the 39 participants that completed the study were as follows (mean ± SD): age, 12.3 ± 0.7 years; height, 155.7 ± 7.5 cm; body mass, 46.0 ± 9.5 kg; body mass index, 18.8 ± 2.6 kg∙m^− 2^; maturity offset, − 1.3 ± 0.7 years.

### Exercise and heart rate

All participants completed the 60 min of games based activity, during which average heart rate was 158 ± 11 beats∙min^− 1^ and maximum heart rate was 197 ± 9 beats∙min^− 1^. There was no difference in average or maximum heart rate during the 60 min basketball between the high and low fitness groups (average, *p* = 0.309; maximum, *p* = 0.177).

### Cognitive function

The data for each of the cognitive function tests at each time point across the exercise and resting trials, for the high and low fitness adolescents, can be found in Table 1. For clarity, cognitive data in figures are presented as change across the morning, given that there were no baseline differences in response time or accuracy between the exercise and resting trials for any test or test level, including when considering the high and low fitness groups separately (all *p* > 0.05). For the Stroop test and Sternberg paradigm response times (of correct responses) were log transformed to exhibit the right hand skew typical of human response times. Minimum (<200 ms) and maximum (1500–3000 ms, depending on task complexity) were applied to eliminate any unreasonably fast (anticipatory) or slow responses.

**Table 1:** Cognitive function data across the exercise and resting trials. Data are mean ± S.E.M.

Test	Level	Variable	Participant Group	Resting trial			Exercise trial		
				Pre-exercise	Immediately post-exercise	45 min post-exercise	Pre-exercise	Immediately post-exercise	45 min post-exercise
Stroop test	Simple	Response time [ms]	Overall	886 ± 28	849 ± 34	852 ± 30	885 ± 22	823 ± 25	933 ± 42
			Low Fitness Group	901 ± 37	842 ± 38	861 ± 49	868 ± 33	825 ± 32	980 ± 60
			High Fitness Group	888 ± 51	878 ± 67	872 ± 43	906 ± 27	824 ± 41	852 ± 41
		Accuracy [%]	Overall	99.1 ± 0.4	97.7 ± 0.6	96.2 ± 1.1	97.6 ± 0.9	97.0 ± 1.2	97.9 ± 0.8
			Low Fitness Group	99.2 ± 0.6	98.3 ± 0.8	96.6 ± 2.1	98.7 ± 0.7	97.9 ± 1.0	98.7 ± 0.7
			High Fitness Group	98.7 ± 0.7	97.5 ± 1.1	95.8 ± 1.2	98.3 ± 0.8	98.3 ± 1.0	98.3 ± 0.8
	Complex	Response time [ms]	Overall	1133 ± 37	1118 ± 31	1140 ± 31	1187 ± 38	1114 ± 29	1124 ± 35
			Low Fitness Group	1164 ± 49	1190 ± 46	1179 ± 45	1131 ± 45	1102 ± 38	1163 ± 57
			High Fitness Group	1130 ± 63	1064 ± 44	1126 ± 46	1255 ± 69	1147 ± 50	1125 ± 49
		Accuracy [%]	Overall	96.8 ± 0.7	94.6 ± 0.8	92.8 ± 1.3	96.6 ± 1.0	94.2 ± 1.2	93.6 ± 1.3
			Low Fitness Group	96.5 ± 1.1	95.0 ± 1.1	93.8 ± 1.6	97.6 ± 0.9	95.3 ± 1.2	94.1 ± 1.4
			High Fitness Group	96.3 ± 1.1	94.4 ± 1.3	92.6 ± 1.9	96.8 ± 1.2	94.1 ± 1.8	94.1 ± 2.0
Sternberg paradigm	One item	Response time [ms]	Overall	553 ± 15	533 ± 15	519 ± 21	548 ± 15	512 ± 16	523 ± 20
			Low Fitness Group	578 ± 26	562 ± 25	558 ± 38	563 ± 23	526 ± 27	565 ± 35
			High Fitness Group	538 ± 20	522 ± 17	484 ± 21	545 ± 24	505 ± 20	495 ± 21
		Accuracy [%]	Overall	98.6 ± 0.6	97.6 ± 0.6	96.6 ± 1.0	97.9 ± 0.7	95.7 ± 1.4	97.6 ± 0.8
			Low Fitness Group	99.7 ± 0.3	98.6 ± 0.8	98.6 ± 0.6	98.3 ± 0.7	94.8 ± 2.8	99.0 ± 0.6
			High Fitness Group	97.1 ± 1.2	97.1 ± 0.9	94.9 ± 1.9	98.2 ± 0.9	96.3 ± 1.1	97.1 ± 1.6
	Three item	Response time [ms]	Overall	712 ± 20	690 ± 23	681 ± 20	710 ± 22	692 ± 21	687 ± 22
			Low Fitness Group	736 ± 29	689 ± 30	686 ± 30	707 ± 36	702 ± 31	697 ± 32
			High Fitness Group	703 ± 31	707 ± 40	688 ± 29	731 ± 31	705 ± 28	685 ± 34
		Accuracy [%]	Overall	95.8 ± 0.6	96.7 ± 0.7	95.0 ± 0.8	97.3 ± 0.5	94.6 ± 1.0	95.0 ± 1.0
			Low Fitness Group	96.7 ± 0.7	97.4 ± 0.7	94.1 ± 1.3	97.4 ± 0.5	95.8 ± 0.8	94.8 ± 1.4
			High Fitness Group	94.3 ± 1.1	95.8 ± 1.5	95.8 ± 1.3	96.9 ± 1.0	94.5 ± 1.8	95.6 ± 1.2
	Five item	Response time [ms]	Overall	861 ± 23	846 ± 28	839 ± 25	878 ± 27	818 ± 25	832 ± 26
			Low Fitness Group	856 ± 30	863 ± 37	830 ± 35	851 ± 33	801 ± 33	820 ± 29
			High Fitness Group	891 ± 40	840 ± 51	873 ± 42	923 ± 45	854 ± 42	868 ± 45
		Accuracy [%]	Overall	94.2 ± 0.9	92.4 ± 1.5	92.7 ± 1.0	93.7 ± 1.2	92.1 ± 1.2	90.1 ± 1.6
			Low Fitness Group	93.9 ± 1.1	92.2 ± 1.8	93.9 ± 1.3	94.6 ± 1.1	90.3 ± 2.1	90.1 ± 2.5
			High Fitness Group	93.6 ± 1.6	92.3 ± 2.7	91.5 ± 1.9	92.1 ± 2.3	93.8 ± 1.2	90.3 ± 2.2
Trail making test	Number	Time taken [ms]	Overall	4827 ± 143	5093 ± 215	5113 ± 202	4777 ± 154	4878 ± 178	5056 ± 195
			Low Fitness Group	4483 ± 215	4991 ± 343	4806 ± 315	4470 ± 220	4761 ± 287	5242 ± 313
			High Fitness Group	5224 ± 215	5311 ± 343	5536 ± 315	5285 ± 220	5152 ± 287	5075 ± 313
	Number and letter	Time taken [ms]	Overall	6539 ± 308	6333 ± 347	5941 ± 236	6847 ± 272	6498 ± 323	6103 ± 274
			Low Fitness Group	5743 ± 449	5666 ± 511	5663 ± 360	6339 ± 409	5777 ± 480	5940 ± 440
			High Fitness Group	7487 ± 449	7356 ± 511	6477 ± 360	7624 ± 408	7389 ± 480	6495 ± 440
d2 test		Concentration Performance	Overall	384 ± 12	425 ± 11	437 ± 13	390 ± 13	424 ± 12	431 ± 12
			Low Fitness Group	371 ± 19	409 ± 16	418 ± 19	362 ± 19	399 ± 18	410 ± 17
			High Fitness Group	395 ± 19	438 ± 16	456 ± 19	414 ± 19	445 ± 18	452 ± 17
		Processed Targets	Overall	396 ± 13	440 ± 12	456 ± 14	404 ± 13	438 ± 13	447 ± 13
			Low Fitness Group	384 ± 20	422 ± 18	435 ± 20	377 ± 19	409 ± 19	424 ± 19
			High Fitness Group	404 ± 20	453 ± 18	478 ± 20	428 ± 19	463 ± 19	472 ± 19
		Error Percentage	Overall	4.8 ± 1.1	4.8 ± 1.0	5.1 ± 0.9	7.8 ± 1.7	6.1 ± 1.1	6.3 ± 1.1
			Low Fitness Group	7.8 ± 1.5	7.2 ± 1.5	7.1 ± 1.4	12.5 ± 2.4	8.6 ± 1.5	9.0 ± 1.6
			High Fitness Group	1.9 ± 1.5	2.4 ± 1.5	3.1 ± 1.4	3.4 ± 2.4	3.9 ± 1.5	3.9 ± 1.6

#### Stroop test

##### Response times

**Fig. 2:**
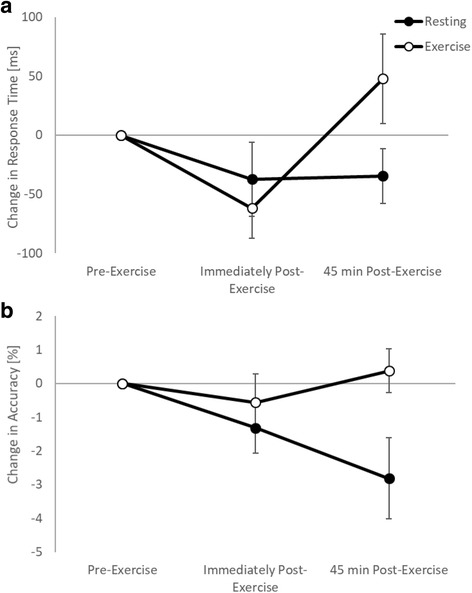
Response times (**a**) and accuracy (**b**) across the morning on the simple level of the Stroop test on the exercise and resting trials

**Fig. 3:**
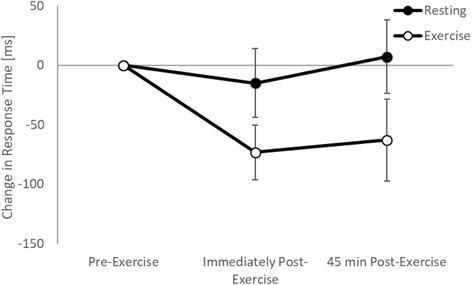
Response times across the morning on the complex level of the Stroop test on the exercise and resting trials

On the simple level of the Stroop test, there was no change in response times immediately following games-based activity (trial by time interaction, *p* = 0.331). However, response times slowed 45 min following games-based activity, whilst they remained similar on the resting trial (trial by time interaction, t_(3287)_ = − 2.6, *p* = 0.009; Fig. 2a). On the complex level of the Stroop test, response times were enhanced both immediately (trial by time interaction, t_(4497)_ = 2.3, *p* = 0.021; Fig. 3) and 45 min (trial by time interaction, t_(4497)_ = 2.3, *p* = 0.035; Fig. 3) following games-based activity on the exercise trial, whilst they remained similar across the morning on the resting trial.

**Fig. 4:**
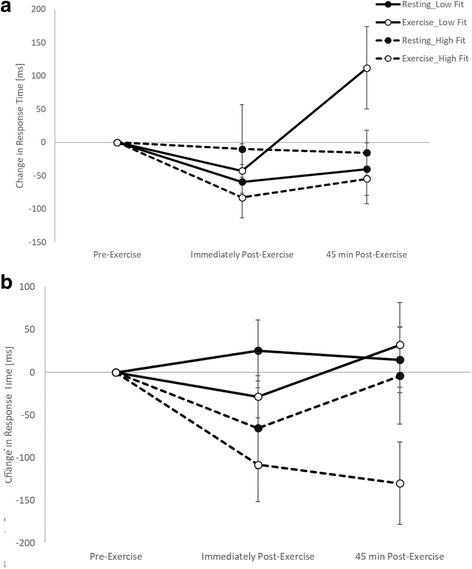
Response times across the morning on the simple (**a**) and complex (**b**) levels of the Stroop test on the exercise and resting trials, with the data split by fitness level

When considering the moderating effect of fitness, on the simple level of the Stroop test there was no difference in the effect of exercise on response times immediately post-exercise between the high and low fit groups (trial by time by fitness interaction, *p* = 0.132). However, 45 min post-exercise whilst response times improved following exercise in the high fitness group, there was a slowing of response times in the low fitness group (trial by time by fitness interaction, t_(2890)_ = 2.8, *p* = 0.005; Fig. 4a). Similar to the baseline level, on the complex level of the Stroop test there was no moderating effect of fitness on response times immediately post-exercise (trial by time by fitness interaction, *p* = 0.565). However, 45 min post-exercise whilst response times improved following exercise in the high fitness group, there was a slowing of response times in the low fitness group (trial by time by fitness interaction, t_(3953)_ = 2.1, *p* = 0.040; Fig. 4b).

##### Accuracy

On the simple level of the Stroop test, there was no change in accuracy immediately following games-based activity (trial by time interaction, *p* = 0.186). However, whilst accuracy was maintained 45 min following games-based activity, there was a decline in accuracy on the resting trial (trial by time interaction, z_(3414)_ = − 2.5, *p* = 0.013; Fig. 2b). On the complex level of the Stroop test, accuracy was unaffected immediately (trial by time interaction, *p* = 0.709) and 45 min (trial by time interaction, *p* = 0.440) following games-based activity.

There was also no moderating effect of fitness on the effects of exercise on accuracy either immediately (trial by time by fitness interactions; simple level: *p* = 0.839; complex level: *p* = 0.701) or 45 min (trial by time by fitness interactions; simple level: *p* = 0.787; complex level: *p* = 0.429) post-exercise.

#### Sternberg paradigm

##### Response times

**Fig. 5:**
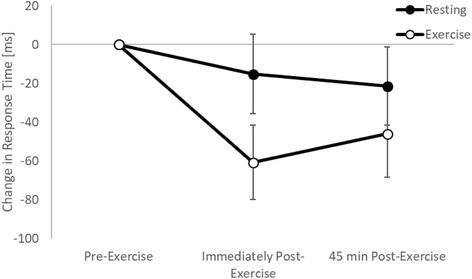
Response times across the morning on the five item level of the Sternberg paradigm on the exercise and resting trials

On the one item and three item levels of the Sternberg paradigm response times were unaffected both immediately (trial by time interactions: one item, *p* = 0.147; three item, *p* = 0.511) and 45 min (trial by time interactions: one item, *p* = 0.480; three item, *p* = 0.501) following games-based activity. On the five item level of the Sternberg paradigm, response times were enhanced immediately following games-based activity whilst remaining unaffected on the resting trial (trial by time interaction, t_(6745)_ = 2.3, *p* = 0.023; Fig. 5). However, this effect did not remain 45 min post-exercise (trial by time interaction, *p* = 0.348).

**Fig. 6:**
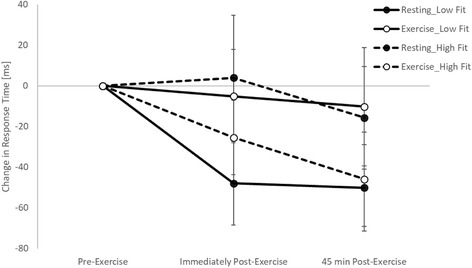
Response times across the morning on the three item level of the Sternberg paradigm on the exercise and resting trials, with the data split by fitness level

When considering the moderating effect of fitness, there was no difference in the effect of exercise on response times between the high and low fit groups on the one item (trial by time by fitness interactions; immediately post-exercise: *p* = 0.940; 45 min post-exercise: *p* = 0.457) or five item (trial by time by fitness interactions; immediately post-exercise: *p* = 0.339; 45 min post-exercise: *p* = 0.295) levels of the Sternberg paradigm. However, on the three item level of the Sternberg paradigm whilst response were enhanced both immediately and 45 min post-exercise in the high fitness group, in the low fitness group response times were enhanced at the corresponding time points on the resting trial (trial by time by fitness interactions; immediately post-exercise, t_(6360)_ = 2.40, *p* = 0.017; 45 min post-exercise, t_(6360)_ = 2.65, *p* = 0.008; Fig. 6).

##### Accuracy

Accuracy was unaffected on all levels of the Sternberg paradigm both immediately (trial by time interaction: one item, *p* = 0.657; three item, *p* = 0.293; five item, *p* = 0.807) and 45 min (trial by time interaction: one item, *p* = 0.190; three item, *p* = 0.103; five item, *p* = 0.282) following games-based activity. There was also no moderating effect of fitness on the effects of exercise on accuracy either immediately (trial by time by fitness interactions; one item level: *p* = 0.506; three item level: *p* = 0.752; five item level, *p* = 0.761) or 45 min (trial by time by fitness interactions; one item level: *p* = 0.237; three item level: *p* = 0.337; five item level, *p* = 0.113) post-exercise.

#### Trail making test

The time taken to complete the number and number/letter level of the trail making test was unaffected by the games-based activity (trial by time interactions: number level, *p* = 0.800; number/letter level, *p* = 0.889). There was also no moderating effect of fitness on the effect of exercise on the time taken to complete either level of the test (trial by time by fitness interactions; number level: *p* = 0.177; number/letter level, *p* = 0.894).

#### d2 test of attention

Concentration performance (CP), the number of processed targets (PT) and the error rate (E%) were all unaffected by the games-based activity (trial by time interactions: CP, *p* = 0.560; PT, *p* = 0.149; E%, *p* = 0.159). There was also no moderating effect of fitness the effect of exercise on any outcome variable from the d2 test (trial by time by fitness interactions; CP: *p* = 0.518; PT: *p* = 0.529; E%, *p* = 0.436).

### Mood

Energy, tension and calmness were unaffected by the games-based activity (trial by time interactions: energy, *p* = 0.638; tension, *p* = 0.984; calmness: *p* = 0.291). However, there was a significant trial by time interaction for tiredness (F_(2,60)_ = 3.3, *p* = 0.042, η^2^ = 0.10), whereby tiredness increased immediately following the games-based activity (exercise: 11.2 ± 4.4, resting: 10.1 ± 4.8; t_(38)_ = − 2.4, *p* = 0.021). There was also a significant trial by time interaction for hunger (F_(2,56)_ = 5.2, *p* = 0.009, η^2^ = 0.157), whereby hunger was lower 45 min following games-based activity on the exercise trial compared to the resting trial (exercise: 69 ± 30, resting: 77 ± 24; t_(29)_ = − 1.3, *p* = 0.036).

When considering the moderating effect of fitness, the effect of the 60 min games-based activity on all components of mood, with the exception of energy, was unaffected (trial by time by fitness interactions; tiredness: *p* = 0.568; tension: *p* = 0.551; calmness, *p* = 0.622; hunger: *p* = 0.764; fullness: *p* = 0.361; concentration: *p* = 0.558). For energy, the low fitness group reported an increase in energy immediately and 45 min post-exercise, whilst energy in the high fitness group was unaffected (trial by time by fitness interaction, F_(2,52)_ = 30.0, *p* = 0.027).

## Discussion

The main findings of the present study are that 60 min games-based activity enhanced working memory and executive function in adolescents, whilst having no effect on attention and information processing speed. This was evidenced by enhanced response times on the complex level of the Stroop test (a measure of executive function) both immediately and 45 min post-exercise, alongside an improvement in response times immediately post-exercise on the 5 item level of the Sternberg paradigm (a test of working memory). These improvements in response time were seen alongside no change in accuracy and thus are indicative of an enhancement in that cognitive domain. Conversely, there was no effect of 60 min games-based activity on any outcome measure on the d2 test (a test of attention) or the time taken to complete the trail making test (a measure of information processing speed). These findings suggest that the effect of 60 min games-based activity may be domain specific and that exercise may be more beneficial for more complex cognitive functions such as executive function.

The present study is also the first to examine the moderating effect of fitness on the acute exercise-cognition relationship. The findings suggest that games-based activity may be particularly beneficial for adolescents with higher fitness levels, whilst possibly being detrimental in lower fit adolescents (as evidenced by altered response times on the Stroop test and Sternberg paradigm), an effect that may be driven by the high intensity intermittent activity patterns of the games-based activity being too demanding for the lower fit participants. Finally, 60 min games-based activity also affected mood, with adolescents reporting greater tiredness and less hunger 45 min post-exercise; with lower fit adolescents also reporting higher energy 45 min post-exercise.

The present study is the first to examine the effects of an ecologically valid exercise model (i.e. 60 min games-based activity) on a range of domains of cognitive function in adolescents. Previous literature in the area has typically employed traditional, moderate intensity, exercise models such as running [4] and cycling [6]. Yet it is rare for adolescents to actually engage in such exercise, with games-based activity not only providing a more attractive exercise mode for many adolescents but also more accurately reflecting the high intensity intermittent nature of their typical physical activity [12, 13, 20]. In the present study the domains of working memory and executive function were enhanced following 60 min games-based activity whilst other more simple cognitive domains (e.g. information processing speed) were unaffected. The effects on executive function and working memory scanning speed are of particular interest for scholastic performance given that these domains have been associated with enhanced academic achievement [39]. The findings of the present study are also in line with the suggestion that high intensity intermittent exercise enhances the speed of executive function [5] and evidence from cross-sectional studies that games-based activity is associated with free recall memory [21] and executive function [23] in adolescents.

The beneficial effect of high intensity intermittent games-based activity on executive function in the present study may be explained by the decision-making and social interactions required in games-based activity, which confer a positive effect on complex cognitive functions such as executive function [40, 41]. Previous evidence suggests that the social interaction and cognitive stimulation required for games-based activity are important determinants of neuroplasticity (i.e. the ability of the nervous system to adapt to the environment) [42], and thus may explain the beneficial effects on executive function in the present study. Furthermore, the social interactions involved in games-based activity may result in the activity being an attractive exercise model for young people, given that social interactions are a key determinant of enjoyment in team games [19].

The present study also extends previous literature by considering the time course of the effects of an acute bout of exercise on cognitive function. Many previous studies only tested at one time point, typically immediately, post-exercise [21, 22]. However, during a school day the more prolonged effects of exercise are of interest, with the time course of the changes important for the structuring of opportunities for exercise during the school day. The findings of the present study suggest that executive function (assessed by the Stroop test) was enhanced immediately and 45 min post-exercise, whereas working memory (assessed by the Sternberg paradigm) was only enhanced immediately post-exercise. This further illustrates that not only are the effects of exercise domain specific, but also that the time course of the changes may be different for different domains. The enhancement of executive function up to 45 min post-exercise will be of particular interest to school policy makers, given its importance for academic achievement [39]. Further research should continue to examine a range of domains of cognition and continue testing beyond 45 min post-exercise to examine how long the beneficial effects remain post-exercise.

Cross-sectional evidence suggests that enhanced memory [25, 26], attention [27] and executive function [7, 26] are associated with higher fitness levels in adolescents. One study in adults also suggests that an acute bout of exercise may be particularly beneficial for cognition in individuals with higher activity and fitness levels [29]. However, the present study is the first to examine this phenomenon in adolescents and suggests that games-based activity was particularly beneficial for adolescents with higher fitness levels (as evidenced by enhanced response times on the Stroop test and Sternberg paradigm). Whilst the exact mechanisms for these effects warrant further investigation, it is possible that the games-based activity in the present study, consisting of high intensity intermittent activity patterns, was too physically demanding for the lower fit adolescents. Whilst there was no difference in heart rate or self-report tiredness between the high and low fitness groups during the basketball, it is likely that the higher fit adolescents are more accustomed to such exercise whilst adolescents with lower fitness levels were not and thus found it more challenging. This suggestion is in line with a meta-analysis showing that high intensity maximal exercise can be detrimental to cognition [43]. The present study adds to this suggestion and furthers that this may only be the case in lower fit adolescents, whereas higher fit adolescents receive cognitive benefits from such exercise. Taken together, this would suggest that exercise should be appropriate to fitness level and that for adolescents to gain maximal cognitive benefits they need to be accustomed to the exercise (and thus regular opportunities for such exercise are important).

The present study has a number of strengths in that it considers the effects of ecologically valid high-intensity intermittent games-based activity and is the first to consider the moderating effect of fitness on the acute exercise-cognition relationship in adolescents. A further strength is that the present study considers the effects of games-based activity across a range of domains of cognition, with particular benefits for more complex cognitive functions such as executive function. However, there are also a number of limitations of the present study such as the indirect assessment of maturity status from sitting height measurements and the use of the multi-stage fitness test to predict VO_2_ peak. Future studies could extend this work by examining whether the effects of exercise on cognition are different across adolescence and/or affected by sexual maturity. Furthermore, to more fully determine the effects of different exercise modalities on neuroplasticity and cognition in adolescents, future studies should compare different types and intensities of exercise (e.g. high-intensity intermittent games-based activity vs. moderate intensity running/cycling), to allow detailed recommendations to be made regarding the use of exercise to enhance adolescents’ cognitive function and thus, academic achievement.

## Conclusions

Overall, the findings of the present study provide novel evidence that games-based activity has beneficial effects on cognitive function up to 45 min post-exercise in adolescents, as demonstrated by improvements in the key cognitive domains of executive function and working memory. The present study also extends previous literature by considering the moderating effect of fitness on the acute exercise-cognition relationship, suggesting that high intensity intermittent games-based activity is particularly beneficial in higher fit adolescents. This suggests that it is important for exercise opportunities in adolescents to be appropriate to the fitness level of the individuals and for adolescents to be regularly exposed to such exercise interventions to ensure they are accustomed to them and thus gain maximal cognitive benefits.
